# Apparent diffusion coefficient histogram analysis can evaluate radiation-induced parotid damage and predict late xerostomia degree in nasopharyngeal carcinoma

**DOI:** 10.18632/oncotarget.19602

**Published:** 2017-07-26

**Authors:** Nan Zhou, Tingting Guo, Huanhuan Zheng, Xia Pan, Chen Chu, Xin Dou, Ming Li, Song Liu, Lijing Zhu, Baorui Liu, Weibo Chen, Jian He, Jing Yan, Zhengyang Zhou, Xiaofeng Yang

**Affiliations:** ^1^ Department of Radiology, Nanjing Drum Tower Hospital, The Affiliated Hospital of Nanjing University Medical School, Nanjing 210008, China; ^2^ Department of Radiology, Nanjing Drum Tower Hospital, Clinical College of Traditional Chinese and Western Medicine, Nanjing University of Chinese Medicine, Nanjing 210008, China; ^3^ The Comprehensive Cancer Centre of Nanjing Drum Tower Hospital, Clinical College of Nanjing Medical University, Nanjing 210008, China; ^4^ Philips Healthcare, Shanghai 200233, China; ^5^ Department of Radiation Oncology and Winship Cancer Institute, Emory University, Atlanta, Georgia 30322, USA

**Keywords:** apparent diffusion coefficient, histogram analysis, radiotherapy, parotid glands, nasopharyngeal carcinoma

## Abstract

We investigated apparent diffusion coefficient (ADC) histogram analysis to evaluate radiation-induced parotid damage and predict xerostomia degrees in nasopharyngeal carcinoma (NPC) patients receiving radiotherapy. The imaging of bilateral parotid glands in NPC patients was conducted 2 weeks before radiotherapy (time point 1), one month after radiotherapy (time point 2), and four months after radiotherapy (time point 3). From time point 1 to 2, parotid volume, skewness, and kurtosis decreased (*P* < 0.001, = 0.001, and < 0.001, respectively), but all other ADC histogram parameters increased (all *P* < 0.001, except *P* = 0.006 for standard deviation [SD]). From time point 2 to 3, parotid volume continued to decrease (*P* = 0.022), and SD, 75^th^ and 90^th^ percentiles continued to increase (*P* = 0.024, 0.010, and 0.006, respectively). Early change rates of parotid ADC_mean_, ADC_min_, kurtosis, and 25^th^, 50^th^, 75^th^, 90^th^ percentiles (from time point 1 to 2) correlated with late parotid atrophy rate (from time point 1 to 3) (all *P* < 0.05). Multiple linear regression analysis revealed correlations among parotid volume, time point, and ADC histogram parameters. Early mean change rates for bilateral parotid SD and ADC_max_ could predict late xerostomia degrees at seven months after radiotherapy (three months after time point 3) with AUC of 0.781 and 0.818 (*P* = 0.014, 0.005, respectively). ADC histogram parameters were reproducible (intraclass correlation coefficient, 0.830 - 0.999). ADC histogram analysis could be used to evaluate radiation-induced parotid damage noninvasively, and predict late xerostomia degrees of NPC patients treated with radiotherapy.

## INTRODUCTION

Xerostomia is a common complication of radiotherapy in patients with nasopharyngeal carcinoma (NPC), which can cause difficult swallowing, tooth decay, and even sleep disorders [1]. Intensity-modulated radiotherapy (IMRT) has been used in NPC patients to spare adjacent normal structures [2, 3], but parotid glands are sensitive to radiation [4], and NPC patients still suffer from xerostomia [2]. An objective evaluation of radiation-induced parotid damage and early prediction of late xerostomia degrees could provide a significant advantage in performing timely intervention to avoid long-term xerostomia.

Xerostomia degrees can be clinically evaluated according to the Radiation Therapy Oncology Group (RTOG) radiation morbidity scoring criteria [5], which is subjective and susceptible to observer variability [6]. Histopathological examination can evaluate the microstructure changes of irradiated parotid glands, which involves an invasive procedure [7]. Scintigraphy can quantitatively assess the functional changes of irradiated parotid glands, but this technique involves extra radiation exposure [8]. Magnetic resonance (MR) imaging, however, can be an objective, noninvasive, and effective tool in evaluating radiation-induced parotid damage [9]. Besides traditional sequences, MR sialography, dynamic contrast-enhanced, diffusion-weighted (DW), intravoxel incoherent motion (IVIM), and T1rho MR imaging have been successfully attempted to evaluate the changes of radiation-induced parotid damage [10–15]. For instance, state of the art recent diffusion imaging uses IVIM, so that the real diffusion of diffusivity (D) can be distinguished from the vascular contamination (D*), providing also additional information on the bloodflow and blood volume (perfusion).

Among those functional modalities, DW imaging proved useful and convenient in evaluating radiation-induced parotid damage [12, 14]. Apparent diffusion coefficient (ADC) values derived from DW imaging could be used to assess the functional changes of irradiated parotid glands [16, 17]. Parotid ADC can reflect the acinar loss and regeneration of irradiated parotid glands, and monitor the long-term radiation-induced parotid damage [18]. However, only mean ADC value obtained from one or several regions of interest (ROIs) was adopted in most previous studies, which might introduce sampling error and neglect the heterogeneity of parotid glands.

ADC histogram analysis provides a series of parameters that can reflect tissue heterogeneity and distribution of ADC values based on pixel distribution. ADC histogram analysis has been successfully used for differential diagnosis, histological differentiation, and assessing therapeutic response in various tumors [19–22]. We detected the dynamic microstructural changes of parotid glands with whole-volume ADC histogram parameters before and after radiotherapy, and assessed if those parameters could predict late xerostomia degrees of NPC patients.

## RESULTS

### Dynamic changes of parotid volume and ADC histogram parameters

Parotid volume decreased from time point 1 to 2 (atrophy rate, 27.24 ± 10.22%; *P* < 0.001), and continued to decrease from time point 2 to 3 (atrophy rate, 14.33 ± 14.79%; *P* = 0.022) (Table 1). Parotid skewness and kurtosis decreased (*P* = 0.001, and < 0.001, respectively), and all the other ADC histogram parameters increased from time point 1 to 2 (all *P* < 0.001, except *P* = 0.006 for SD), and SD, 75^th^, and 90^th^ percentiles continued to increase from time point 2 to 3 (*P* = 0.024, 0.010, and 0.006, respectively).

**Table 1:** Dynamic changes of parotid volume and apparent diffusion coefficient (ADC) histogram parameters during radiotherapy in patients with nasopharyngeal carcinoma

Parameters	Time point 1	Time point 2	Time point 3	*P* value
Volume	27.15 ± 6.53	19.47 ± 4.38*	16.60 ± 4.56*^,§^	< 0.001
ADC_mean_	726.5 ± 79.2	1084.6 ± 140.5*	1136.5 ± 187.0*	< 0.001
SD	177.3 ± 31.0	195.2 ± 34.2*	209.9 ± 36.8*^,§^	< 0.001
ADC_min_	132.4 ± 127.5	426.1 ± 274.8*	476.8 ± 292.0*	< 0.001
ADC_max_	1521.5 ± 194.2	1811.8 ± 223.3*	1882.8 ± 202.6*	< 0.001
5^th^ percentile	449.3 ± 119.7	780.7 ± 164.2*	799.5 ± 230.3*	< 0.001
10^th^ percentile	514.4 ± 104.2	847.3 ± 154.1*	875.4 ± 210.8*	< 0.001
25^th^ percentile	613.8 ± 86.2	955.1 ± 142.1*	996.5 ± 193.4*	< 0.001
50^th^ percentile	719.9 ± 75.9	1078.9 ± 140.9*	1130.6 ± 187.0*	< 0.001
75^th^ percentile	831.7 ± 72.4	1205.8 ± 138.5*	1273.9 ± 183.2*^,§^	< 0.001
90^th^ percentile	943.2 ± 76.1	1331.9 ± 140.1*	1404.1 ± 173.6*^,§^	< 0.001
Skewness	0.781 ± 0.806	0.308 ± 0.689*	0.418 ± 0.748*	0.003
Kurtosis	9.718 ± 2.609	6.815 ± 2.687*	7.120 ± 2.575*	< 0.001

Note: ADC_mean_, ADC_min_, and ADC_max_ are the mean, minimum, and maximum ADC values of all voxels within the volume of interest, respectively. SD is the standard deviation of ADC values. The 5^th^, 10^th^, 25^th^, 50^th^, 75^th^, and 90^th^ percentiles are the ADC values at which 5%, 10%, 25%, 50%, 75%, and 90% of the voxel values that formed the histogram are found to the left, respectively. Skewness is the degree of histogram asymmetry around the mean. Kurtosis is a measurement of the histogram sharpness. Time point 1 was within 2 weeks before radiotherapy. Time point 2 was one month after radiotherapy. Time point 3 was four months after radiotherapy. *P* values were calculated by one-way analysis of variance.

The volume unit is cm^3^, and all the ADC histogram parameters are in the units of × 10^-6^ mm^2^/s except for skewness and kurtosis.

* *P* < 0.05 compared to time point 1.

^§^ *P* < 0.05 compared to time point 2.

**Figure 1:**
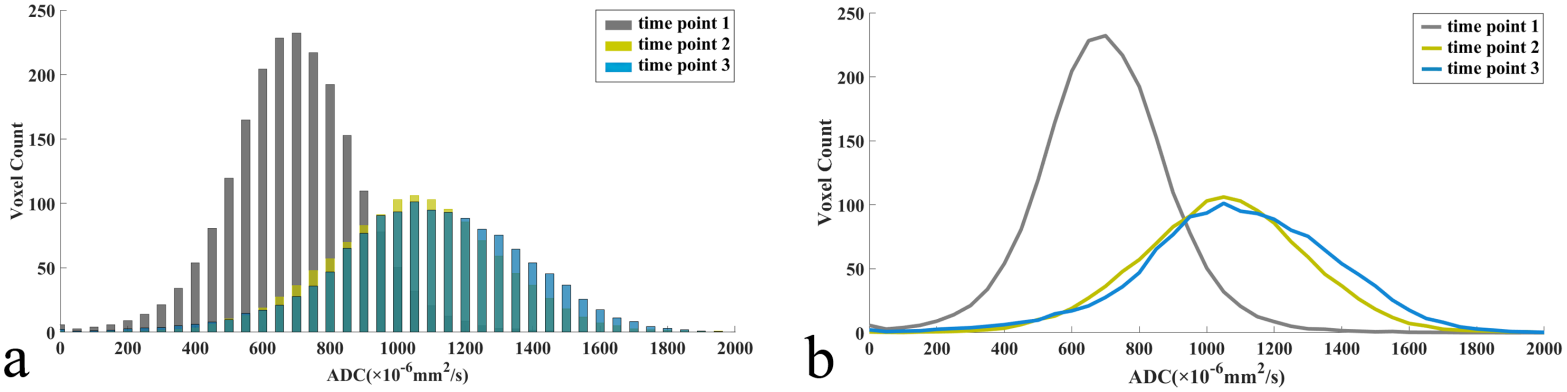
Dynamic changes of mean apparent diffusion coefficient (ADC) histograms **(a)**, (bin size of 50 × 10^-6^ mm^2^/s) and its corresponding fitting curves **(b)** of 56 parotid glands in 28 patients with nasopharyngeal carcinoma receiving radiotherapy.

Mean ADC histogram shifted to the right with an increased width and a decreased peak from time point 1 to 3 (Figure 1). Early change rates of parotid ADC_mean_, ADC_min_, kurtosis, and 25^th^, 50^th^, 75^th^, 90^th^ percentiles from time point 1 to 2 correlated with late parotid atrophy rate from time point 1 to 3 (all *P* < 0.05; Table 2). There were no correlations between the change rates of parotid ADC histogram parameters from time point 1 to 2 or 3 and mean radiation dose (all *P* > 0.05). No differences of mean change rates of bilateral parotid ADC histogram parameters were found between T1/2 and T3/4 stages from time point 1 to 2 or 3 (all *P* > 0.05).

**Table 2:** Correlations between early change rates of parotid apparent diffusion coefficient (ADC) histogram parameters and late parotid atrophy rate in patients with nasopharyngeal carcinoma

	Early change rate (from time point 1 to 2)	Correlation coefficient (r)	*P* value
Late atrophy rate (from time point 1 to 3)	ADC_mean_	0.484	0.003*
	SD	− 0.187	0.275
	ADC_min_	0.477	0.016*
	ADC_max_	0.047	0.784
	5^th^ percentile	0.219	0.198
	10^th^ percentile	0.319	0.058
	25^th^ percentile	0.430	0.009*
	50^th^ percentile	0.475	0.003*
	75^th^ percentile	0.481	0.003*
	90^th^ percentile	0.489	0.002*
	Skewness	− 0.141	0.413
	Kurtosis	− 0.380	0.022*

Note: ADC_mean_, ADC_min_, and ADC_max_ are the mean, minimum, and maximum ADC values of all voxels within the volume of interest, respectively. SD is the standard deviation of ADC values. The 5^th^, 10^th^, 25^th^, 50^th^, 75^th^, and 90^th^ percentiles are the ADC values at which 5%, 10%, 25%, 50%, 75%, and 90% of the voxel values that formed the histogram are found to the left, respectively. Skewness is the degree of histogram asymmetry around the mean. Kurtosis is a measurement of the histogram sharpness. Time point 1 was within 2 weeks before radiotherapy. Time point 2 was one month after radiotherapy. Time point 3 was four months after radiotherapy.

* Significant correlations with Pearson correlation test.

### Correlations among parotid volume, time point and ADC histogram parameters

The parotid ADC_mean_, ADC_min_, ADC_max_, 5^th^, 10^th^, 25^th^, 50^th^, 75^th^, and 90^th^ percentiles negatively correlated with parotid volume, but positively correlated with MR scan time point (radiotherapy course). With the exception of ADC_min_, MR scan time point showed a higher influence (with larger standardized coefficients) than parotid volume on those ADC histogram parameters (Table 3). Parotid SD and skewness were only associated with MR scan time point and independent from parotid volume. However, kurtosis was only associated with parotid volume.

**Table 3:** Multiple linear regression analysis of volume and MR scan time point contributing to apparent diffusion coefficient (ADC) histogram parameters

Parameters	R^2^	Volume		Time point	
		Standardized coefficients	*P* value	Standardized coefficients	*P* value
ADC_mean_	0.564	-0.269	0.001*	0.555	< 0.001*
SD	0.140	-0.043	0.703	0.346	0.002*
ADC_min_	0.280	-0.308	0.003*	0.281	0.007*
ADC_max_	0.391	-0.256	0.007*	0.434	< 0.001*
5^th^ percentile	0.404	-0.289	0.002*	0.415	< 0.001*
10^th^ percentile	0.464	-0.289	0.001*	0.464	< 0.001*
25^th^ percentile	0.524	-0.269	0.002*	0.527	< 0.001*
50^th^ percentile	0.560	-0.255	0.002*	0.564	< 0.001*
75^th^ percentile	0.601	-0.257	0.001*	0.590	< 0.001*
90^th^ percentile	0.621	-0.262	0.001*	0.599	< 0.001*
Skewness	0.047	-0.128	0.277	0.270	0.024*
Kurtosis	0.235	0.432	< 0.001*	-0.080	0.448

Note: ADC_mean_, ADC_min_, and ADC_max_ are the mean, minimum, and maximum ADC values of all voxels within the volume of interest, respectively. SD is the standard deviation of ADC values. The 5^th^, 10^th^, 25^th^, 50^th^, 75^th^, and 90^th^ percentiles are the ADC values at which 5%, 10%, 25%, 50%, 75%, and 90% of the voxel values that formed the histogram are found to the left, respectively. Skewness is the degree of histogram asymmetry around the mean. Kurtosis is a measurement of the histogram sharpness. Time point 1 was within 2 weeks before radiotherapy. Time point 2 was one month after radiotherapy. Time point 3 was four months after radiotherapy.

* Significant correlations with multiple linear regression analysis.

### Greater ADC_max_ correlates to grade 2 xerostomia

Early mean change rates of bilateral parotid SD and ADC_max_ were larger in patients with grade 2 xerostomia (SD, 22.7 ± 15.3%; ADC_max_, 27.7 ± 10.3%) than those with grade 1 (SD, 6.8 ± 15.6%; ADC_max_, 14.8 ± 13.4%) at seven months after radiotherapy (*P* = 0.014, 0.008, respectively). With cutoffs of 10.8% and 16.0%, early mean change rates of SD and ADC_max_ showed sensitivity of 90.9% and 100.0%, specificity of 70.6% and 70.6%, respectively, in differentiating patients with grade 2 xerostomia from those with grade 1 at seven months after radiotherapy (Table 4).

**Table 4:** Diagnostic performance of early mean change rates of bilateral parotid volume and apparent diffusion coefficient (ADC) histogram parameters in predicting xerostomia degrees in patients with nasopharyngeal carcinoma seven months after radiotherapy

Change rates (from time point 1 to 2)	Cutoff value (%)	Sensitivity (%)	Specificity (%)	AUC (95% CI)	*P* value
Volume	26.6	81.8	28.6	0.766 (0.539 - 0.993)	0.063
ADC_mean_	61.0	54.5	88.2	0.588 (0.328 - 0.849)	0.438
SD	10.8	90.9	70.6	0.781 (0.593 - 0.969)	0.014*
ADC_min_	128.2	77.8	62.5	0.708 (0.449 - 0.968)	0.149
ADC_max_	16.0	100.0	70.6	0.818 (0.656 - 0.980)	0.005*
5^th^ percentile	37.2	94.1	36.4	0.524 (0.270 - 0.778)	0.832
10^th^ percentile	45.0	88.2	54.5	0.513 (0.255 - 0.772)	0.906
25^th^ percentile	74.5	36.4	94.1	0.551 (0.299 - 0.803)	0.655
50^th^ percentile	63.4	54.5	94.1	0.604 (0.346 - 0.862)	0.359
75^th^ percentile	56.1	54.5	94.1	0.615 (0.362 - 0.868)	0.312
90^th^ percentile	49.1	54.5	88.2	0.599 (0.347 - 0.851)	0.384
Skewness	-12.7	27.3	94.1	0.503 (0.256 - 0.750)	0.981
Kurtosis	-14.1	54.5	88.2	0.679 (0.460 - 0.899)	0.115

Note: AUC is the area under receiver operating characteristic (ROC) curve. ADC_mean_, ADC_min_, and ADC_max_ are the mean, minimum, and maximum ADC values of all voxels within the volume of interest. SD is the standard deviation of ADC values. The 5^th^, 10^th^, 25^th^, 50^th^, 75^th^, and 90^th^ percentiles are the ADC values at which 5%, 10%, 25%, 50%, 75%, and 90% of the voxel values that formed the histogram are found to the left, respectively. Skewness is the degree of histogram asymmetry around the mean. Kurtosis is a measurement of the histogram sharpness. Time point 1 was within 2 weeks before radiotherapy. Time point 2 was one month after radiotherapy. CI: confidence interval.

* *P* < 0.05.

### Reproducibility of ADC histogram parameter measurements

The inter- and intra-observer agreements of all ADC histogram parameters were excellent (ICCs, 0.830-0.999; Table 5).

**Table 5:** Inter- and intra-observer agreements of apparent diffusion coefficient (ADC) histogram parameters

Parameters	Inter-observer ICC (95% CI)	Intra-observer ICC (95% CI)
ADC_mean_	0.997 (0.995 - 0.998)	0.998 (0.996 - 0.999)
SD	0.908 (0.837 - 0.948)	0.931 (0.881 - 0.960)
ADC_min_	0.947 (0.908 - 0.969)	0.960 (0.929 - 0.977)
ADC_max_	0.857 (0.754 - 0.917)	0.830 (0.701 - 0.904)
5^th^ percentile	0.997 (0.994 - 0.998)	0.998 (0.996 - 0.999)
10^th^ percentile	0.997 (0.996 - 0.999)	0.998 (0.997 - 0.999)
25^th^ percentile	0.998 (0.997 - 0.999)	0.999 (0.998 - 0.999)
50^th^ percentile	0.998 (0.997 - 0.999)	0.998 (0.997 - 0.999)
75^th^ percentile	0.993 (0.988 - 0.996)	0.997 (0.994 - 0.998)
90^th^ percentile	0.992 (0.986 - 0.995)	0.995 (0.993 - 0.996)
Skewness	0.914 (0.852 - 0.950)	0.921 (0.860 - 0.955)
Kurtosis	0.947 (0.909 - 0.969)	0.964 (0.937 - 0.980)

Note: ADC_mean_, ADC_min_, and ADC_max_ are the mean, minimum, and maximum ADC values of all voxels within the volume of interest, respectively. SD is the standard deviation of ADC values. The 5^th^, 10^th^, 25^th^, 50^th^, 75^th^, and 90^th^ percentiles are the ADC values at which 5%, 10%, 25%, 50%, 75%, and 90% of the voxel values that formed the histogram are found to the left, respectively. Skewness is the degree of histogram asymmetry around the mean. Kurtosis is a measurement of the histogram sharpness. ICC: intraclass correlation coefficient; CI: confidence interval.

## DISCUSSION

ADC histogram analysis has been successfully introduced into the evaluation of radiation-induced parotid damage in NPC patients undergoing radiotherapy. We found that parotid volume, skewness, and kurtosis decreased, while all the other ADC histogram parameters increased after radiotherapy. Early mean change rates of certain ADC histogram parameters proved useful in predicting late xerostomia degrees.

Parotid volume decreased from time point 1 to 3, which suggested the widespread degeneration and necrosis of acinar cells [23]. Marzi et al reported a parotid atrophy of 31% after radiotherapy in patients with head and neck cancer [14], which was similar to our findings. It was reported that ADC_mean_ of parotid glands increased after radiotherapy [14, 17], which was consistent with our findings. A possible explanation was that radiation-induced loss of parotid acinar cells caused an enlarged extracellular space and an augmented water molecular diffusion [3].

The increased parotid SD indicated a larger distribution of ADC values. We speculated that the acinar cell necrosis and inflammatory cell infiltration caused more heterogeneous microstructures of irradiated parotid glands, and consequently increased SD. A decrease of skewness was expected as the peak of the histogram shifted from low ADC area toward relatively high ADC area, which could be explained by acinar cell necrosis. A decrease of kurtosis was also expected as the ADC values of parotid gland showed more heterogeneous distribution and a flatter histogram peak [24].

We found that the 75^th^ and 90^th^ percentiles of parotid glands continued to increase from one month to four months after radiotherapy. The tail (higher percentiles) of the mean ADC histogram moved toward right, while the body of the mean ADC histogram did not move. Lower percentiles (5^th^, 10^th^, and 25^th^ percentiles) in the ADC histogram represented regions with dense cells, while higher percentiles (50^th^, 75^th^, and 90^th^ percentiles) corresponded to areas with less restricted water molecular distribution [21]. It could be that the parotid ducts started to be repaired, leading to an increase of the 75^th^ and 90^th^ percentiles. The visibility score of parotid ducts on MR sialography improved at 6 months after radiotherapy compared to 6 weeks after radiotherapy, indicating a repair process of parotid ducts [10].

SD values also increased from time point 2 to 3, indicating more heterogeneity due to fibrosis and repair of parotid glands four months after radiotherapy [23]. Yang et al also reported that the 3-dB bandwidth further widened from acute toxicity (with 3 months after radiotherapy) to late toxicity of parotid glands (beyond 3 months after radiotherapy), indicating a more heterogeneous texture of parotid parenchyma [25].

Early change rates of parotid ADC_mean_, ADC_min_, kurtosis, and 25^th^, 50^th^, 75^th^, 90^th^ percentiles correlated with late parotid atrophy rate, which suggested that ADC histogram analysis could be used to evaluate radiation-induced parotid damage noninvasively. There were no significant correlations between the change rates of parotid ADC histogram parameters and mean radiation dose in this study. Marzi et al also detected no significant correlation between change of mean ADC value and mean radiation dose before and after radiotherapy [14]. However, they found a significant correlation between change in pure diffusion coefficient D and mean radiation dose. A possible explanation is that D represents pure diffusion, while ADC contains information from both pure diffusion and perfusion-related diffusion. This indicated that D was superior to ADC in evaluating radiation-induced parotid damage, and histogram analysis of IVIM parameters, especially D values, deserves further investigation.

Early mean change rates of bilateral parotid SD and ADC_max_ (from time point 1 to 2) were larger in patients with grade 2 xerostomia than those with grade 1 at seven months after radiotherapy. This might demonstrate higher heterogeneity and larger extracellular space of parotid gland in patients with more severe xerosotmia. Those two indices could predict late xerostomia degrees, which might be useful predictive indicators for differentiating between long-term xerostomia degrees in NPC patients treated with radiotherapy.

Although whole volume histogram analysis of radiation-induce parotid damage is a time-consuming process, it can provide valuable information which could not be obtained from conventional approaches. For instance, SD, 75^th^, and 90^th^ percentiles of parotid glands continued to increase from one month to four months after radiotherapy, which might indicate the repair of parotid ducts and the existence of fibrosis. However, simple mean ADC value based on circular ROIs remained unchanged. The heterogeneity of irradiated parotid glands could be quantitatively evaluated by using ADC histogram parameters, which could not be detected by simple mean ADC values. Moreover, the early mean change rates of bilateral parotid SD and ADC_max_ could predict late xerostomia degree, while parotid mean ADC values failed to predict it. The histogram approach proved superior to conventional circular ROIs approach in evaluating microstructural changes of irradiated parotid glands. Additionally, all ADC histogram parameters in our study showed high inter- and intra-observer reproducibility. ADC_max_ might be influenced by some extreme values, which led to a relatively lower inter- and intra-ICC for ADC_max_. But the inter- and intra-observer agreement of ADC_max_ was pretty good with ICCs of 0.857 and 0.830.

A major limitation in our study was that the impact of radiation-induced damage of submandibular glands, sublingual glands, and minor salivary glands was not taken into consideration. Since those glands were relatively or extremely small, ADC histogram parameters of them were difficult to measure. Taking all salivary glands into consideration will be more comprehensive, but the parotid glands are the largest salivary glands, which produce 60%-65% of the whole saliva [26].

Sample size was relatively small. However, we found that the power of all parotid MR parameters for one-way analysis of variance (ANOVA) were all greater than 0.800 and adequate for statistical analysis [27].

There was lack of histopathological findings as reference due to the invasiveness of biopsy procedure. The ADC values were determined by two *b*-values (0 and 1000 s/mm^2^), which could be contaminated by blood perfusion in the gland parenchyma. However, there is precedence for using this method [18, 28]. A follow-up duration of 4-7 months may be too short. Braam et al reported an increase in stimulated flow rate of parotid glands of 42.9% from 6 weeks to 6 months after radiotherapy and 25% from 6 months to 5 years after radiotherapy [29]. We also found that the xerostomia degree in most patients (26/28, 92.9%) remained unchanged from 4 months to 7 months after radiotherapy. Hence, we speculated that changes of xerostomia in patients with NPC became subtle after 6 months of radiotherapy.

In conclusion, most ADC histogram parameters of irradiated parotid glands increased, and early change rates of parotid ADC_mean_, ADC_min_, kurtosis, and 25^th^, 50^th^, 75^th^, 90^th^ percentiles correlated with late parotid atrophy rate. Early mean change rates of parotid SD and ADC_max_ could predict late xerostomia degrees. Therefore, ADC histogram analysis increased our capability to objectively evaluate radiation-induced microstructural changes of parotid glands and predict late xerostomia degrees of NPC patients. This might facilitate early intervention to spare parotid function and avoid long-term xerostomia.

## MATERIALS AND METHODS

### Patients

This study was approved by the institutional review board, and all patients provided written informed consents. From October 2015 to December 2016, 28 patients (25 men, 3 women, age 48.3 ± 13.5 years) with an initial diagnosis of nasopharyngeal squamous cell carcinoma treated with IMRT were prospectively enrolled (Table 6). The eligibility criteria for patients were as follows: (1) ≥ 18 years old, (2) pathological diagnosis of squamous cell carcinoma of nasopharynx, (3) no prior radiotherapy to head and neck, (4) no history of parotid gland diseases such as parotid gland malignancy, (5) no contraindications to MR examination such as artificial cochlea and cardiac pacemaker.

**Table 6:** Characteristics of NPC patients treated with radiotherapy

Characteristics	Value
Total patients (men/women)	28 (25/3)
Mean age (years) age range	48.3 ± 13.5 21 - 68
T stage	
T1	15 (53.6%)
T2	9 (32.1%)
T3	3 (10.7%)
T4	1 (3.6%)
N stage	
N0	2 (7.1%)
N1	8 (28.6%)
N2	16 (57.2%)
N3	2 (7.1%)
Grade 0/1/2 xerostomia (patients)	
Time point 1	28/0/0
Time point 2	0/15/13
Time point 3	0/15/13
Seven months after radiotherapy	0/17/11
Mean radiation dose of parotid glands (Gy)	28.4 ± 2.6

Note: Time point 1 was within 2 weeks before radiotherapy. Time point 2 was one month after radiotherapy. Time point 3 was four months after radiotherapy. Xerostomia degrees were evaluated by the Radiation Therapy Oncology Group (RTOG) acute and late radiation morbidity scoring criteria. The TN staging was performed based on the American Joint Committee on Cancer (AJCC) and the Union for International Cancer Control (UICC). NPC: nasopharyngeal carcinoma.

All patients were treated with IMRT and concurrent chemotherapy of nedaplatin (60 mg each fraction, three fractions with an interval of one week). The total radiation dose to the gross tumor region was 70 Gy, which was divided into 35 fractions over seven weeks. The clinical target volume (CTV) covered the gross tumor region, the neck lymphatic drainage area, and the high-risk regions (e.g. the parapharyngeal space and the base of skull). The planning target volume (PTV) expanded the CTV by 5 millimeters in three dimensions. The radiotherapy plan was optimized to spare the parotid glands. The radiation dose to all parotid glands was below the dose constraint of our hospital, 30-35 Gy for 50% of the volume.

The treatment planning systems of Pinnacle^3^ (Philips Medical Systems, Fitchburg, WI, USA) and TomoTherapy HiArt (TomoTherapy, Madison, WI, USA) were used to formulate the radiotherapy plan. The mean radiation dose of each fraction to the parotid gland was determined by the dose volume histogram (DVH) of the treatment planning systems. The mean radiation doses of bilateral parotid glands were 27.9 ± 3.1 Gy, 28.8 ± 2.3 Gy, and 29.2 ± 1.8 Gy in T1, T2, and T3/4 stages, and 27.9 ± 2.2 Gy, 29.0 ± 2.7 Gy in N0/1 and N2/3 stages, respectively. Although the mean radiation dose tended to increase with T and N stages, no differences could be detected among different T and N stages (*P* = 0.633 and 0.380, respectively). Bilateral parotid glands were analyzed separately because they were exposed to different radiation doses.

MR examinations occurred at three time points: within 2 weeks before radiotherapy (time point 1), one month after radiotherapy (time point 2), and four months after radiotherapy (time point 3). One hour before each MR scan, the xerostomia degree of each patient was evaluated by a radiation oncologist (X.X.) based on the RTOG acute and late radiation morbidity scoring criteria [5]. The late xerostomia degree at seven months after radiotherapy was also evaluated. The acute scoring criteria were: grade 0, no change over baseline; grade 1, slightly thickened saliva without an increased use of liquids with meals; grade 2, sticky saliva accompanied with the usage of liquids with meals; grade 3, complete dryness of mouth without salivation; grade 4, acute salivary gland necrosis. The late scoring criteria: grade 0, no change over baseline; grade 1, slight dryness of mouth without the usage of liquids with meals; grade 2, moderate dryness of mouth accompanied with the usage of liquids with meals; grade 3, complete dryness of mouth without salivation; grade 4, fibrosis.

Based on previous studies associated with DW imaging near the skull base [30] and our clinical experience, DW image quality was evaluated according to the following four factors: physiologic motion, geometric distortion, signal loss and ghosting artifacts. The score was recorded as 1 if one factor existed, 2 if two factors existed, etc, and 0 if none of them existed. The image quality was adequate when the total score was ≤ 1, and we found that the image quality of DW imaging was adequate in all patients in our study (58 sets of DW images scored as 0, and 26 sets scored as 1 for 12 sets with ghosting artifacts, 7 sets with physiologic motion, 5 sets with signal loss and 2 sets with geometric distortion).

### MRI

A full digital 3.0-T MR scanner (Ingenia, Philips Medical Systems, Best, the Netherlands) was used for MR scans, using a16-channel head/neck phased array coil with head-first and supine position. The MR scanning sequences included transverse T1-weighted imaging, transverse DW imaging for bilateral parotid glands, and the regular MR sequences for nasopharynx and neck. Those sequences included T1-weighted imaging without contrast in transverse, coronal, and sagittal planes, T2-weighted imaging with fat suppression in transverse and coronal planes, and T1-weighted imaging with contrast and fat suppression in transverse, coronal, and sagittal planes.

The parameters of sequences for bilateral parotid glands are as follows. T1-weighted imaging was obtained with a multi-shot turbo spin-echo sequence (repetition time = 400-675 msec, echo time = 18 msec, slices = 12, slice thickness = 4 mm, slice gap = 1 mm, field of view = 24 cm, voxel size = 0.8 mm × 0.91 mm, matrix = 300 × 245, number of signal averaged = 2). The scan duration was 45 s.

DW imaging was obtained with a single-shot turbo spin-echo (SS-TSE) sequence to reduce susceptibility artifacts at the skull base [31]. The sequence contained *b* values = 0, 1000 s/mm^2^, repetition time = 4569 msec, echo time = 71 msec, slices = 12, slice thickness = 4 mm, slice gap = 1 mm, field of view = 24 cm, voxel size = 1.8 mm × 2.0 mm, matrix = 132 × 120, number of signal averaged = 4, and a 3 min 02 s scan duration.

### Image analyses

All MR images were independently analyzed by two radiologists (X.X. and X.X.X.) that were blinded to clinical patient information, and their measurements were averaged. To analyze the intra-observer reproducibility, parotid ADC histogram parameters were repeatedly measured by the second observer with an interval of 6 weeks. In our pilot study, we attempted to apply a Gaussian filter to remove the noise in image preprocessing, but we found it made no difference on histogram parameters (all *P* values > 0.05). Down-sampling made the images appear blurred, so ADC histogram parameters were analyzed by using our raw ADC images. The power of sample size for one-way ANOVA was calculated by using G* Power software version 3.1.2 (Franz Faul, Universitat Kiel, Germany) [32], and the power of parotid MR parameters was listed in Table 7.

**Table 7:** Power of simple size for one-way analysis of variance (ANOVA)

Parameters	Power
Volume	0.972
ADC_mean_	1.000
SD	0.956
ADC_min_	0.979
ADC_max_	0.976
5^th^ percentile	0.995
10^th^ percentile	0.985
25^th^ percentile	0.999
50^th^ percentile	0.954
75^th^ percentile	0.980
90^th^ percentile	0.978
Skewness	0.953
Kurtosis	0.951

Note: ADC_mean_, ADC_min_, and ADC_max_ are the mean, minimum, and maximum ADC values of all voxels within the volume of interest, respectively. SD is the standard deviation of ADC values. The 5^th^, 10^th^, 25^th^, 50^th^, 75^th^, and 90^th^ percentiles are the ADC values at which 5%, 10%, 25%, 50%, 75%, and 90% of the voxel values that formed the histogram are found to the left, respectively. Skewness is the degree of histogram asymmetry around the mean. Kurtosis is a measurement of the histogram sharpness.

Parotid volume was calculated on a workstation (Extended MR WorkSpace 2.6.3.5, Philips Medical Systems, Best, the Netherlands) by using equation 1:

$$\mathrm{V=}\sum S_{i}\times \left(\mathrm{ST+SG}\right)$$ (1)

where V is parotid volume, S_*i*_ is the area of the *i*th slice, ST is the slice thickness and SG is the slice gap. The change rates of parotid volume from time point 1 to 2 and 3 were defined as parotid atrophy rates and calculated by using equation 2:

$$R_{V}=\left(V_{1}-V_{2/3}\right)/V_{1}\mathrm{\times 100\%}$$ (2)

where R_V_ represents the parotid atrophy rate at time point 2 or 3 compared with time point 1, and V_1_ and V_2/3_ represent the parotid volume at time point 1, 2 and 3, respectively.

**Figure 2:**
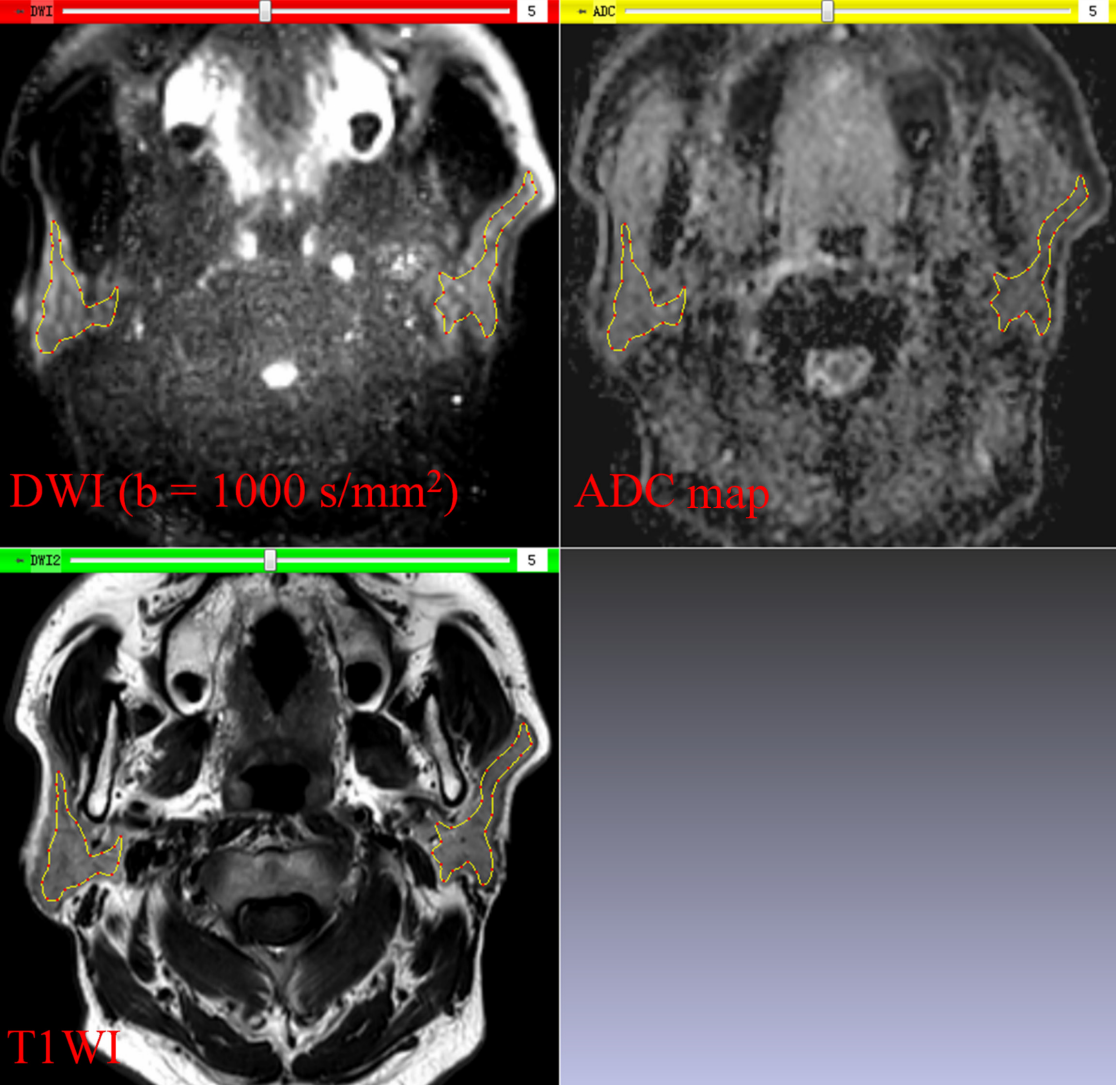
In-house software screenshot The region of interest is exactly copied on the diffusion weighted image (DWI) to the corresponding T1-weighted image (T1WI) and apparent diffusion coefficient (ADC) map in real time. Parotid glands can be distinguished from the adjacent structures on DWI and T1WI.

The ADC maps were generated from DW images using a mono-exponential model on the workstation. The whole-volume ADC histogram analysis of 56 parotid glands from 28 patients was performed using an in-house software (Image Analyzer 1.0, China). The workflow of the histogram analysis was described as follows:

(1) ADC maps, the corresponding DW images, and T1-weighted images were loaded together into our in-house software.
(2) Each ROI was drawn manually along the inner margin of the parotid glands on DW images, excluding visible retromandibular veins. Since DW images of parotid glands were acquired with the same slice thickness, slice gap, and field of view as T1-weighted images, the ROIs could be shown on the corresponding T1-weighted images and ADC maps in real time (Figure 2).
(3) After delineation of all ROIs that covered the whole volume of parotid gland, a set of ADC histogram parameters were generated. Those parameters including: (a) ADC_mean_, the mean value of all ADC values within the volume of interest (VOI); (b) ADC_min_, the minimum value of all ADC values within the VOI; (c) ADC_max_, the maximum value of all ADC values within the VOI; (d) SD, the standard deviation of all ADC values within the VOI; (e) 5^th^, 10^th^, 25^th^, 50^th^, 75^th^, and 90^th^ percentiles, the ADC values at which 5%, 10%, 25%, 50%, 75%, and 90% of the voxel values that formed the histogram are found to the left, respectively; (f) skewness, the degree of histogram asymmetry around the mean; (g) kurtosis, a measurement of the histogram sharpness.

The change rates of parotid ADC histogram parameters from time point 1 to time point 2 and 3 were calculated by using equation 3:

$$R_{\mathrm{PAR}}=\left(\mathrm{PA}R_{2/3}-\mathrm{PA}R_{1}\right)/\mathrm{PA}R_{1}\times 100\%$$ (3)

where R_PAR_ is the change rate of ADC histogram parameters from time point 1 to time point 2 and 3, PAR_1_ and PAR_2/3_ are the ADC histogram parameters at time point 1, time point 2 and 3, respectively.

### Generating mean ADC histogram

To visually observe the dynamic changes of ADC histogram, the mean ADC histograms and its corresponding fitting curves at each time point were generated using Matlab software (Matlab, R2010b; Mathworks, Natick, Mass). At each time point, we divided the ADC values of each patient into a series of isometric intervals with a bin size of 50 × 10^-6^ mm^2^/s. We calculated the mean ADC frequency in the same interval of the 56 parotid glands. The ADC intervals and their corresponding mean frequency were loaded into the software to create the mean ADC histograms and its corresponding fitting curves.

### Statistical analyses

Continuous quantitative data with normal distribution were shown as mean ± standard deviation. Changes of volume and ADC histogram parameters from time point 1 to 3 was compared using one-way ANOVA. Least significant difference method was adopted for further comparison between each time point. The Pearson correlation test assessed the change rates of parotid ADC histogram parameters and mean radiation dose, and between the early change rates of ADC histogram parameters and late atrophy rate. Multiple linear regression analysis was performed to explore the correlations among parotid volume, MR scan time point, and ADC histogram parameters. Independent-samples *t*-test was used to analyze the difference of the early mean change rates of ADC histogram parameters in bilateral parotid glands from time point 1 to 2 between grade 1 and grade 2 xerostomia degrees at seven months after radiotherapy. The diagnostic performance of those early mean change rates in predicting late xerostomia degrees was evaluated with receiver operating characteristic (ROC) analysis. Wilcoxon rank sum test was used to analyze the effect of tumor staging on the changes of ADC histogram parameters. Intraclass correlation coefficient (ICC) was used to analyze the reproducibility of ADC histogram parameters. The statistical analyses were performed using SPSS 16.0 software (SPSS Inc., Chicago, IL). Two-sided *P* values < 0.05 were considered statistically significant.

## Abbreviations

ADC: apparent diffusion coefficient
NPC: nasopharyngeal carcinoma
SD: standard deviation
IMRT: intensity-modulated radiotherapy
MR: magnetic resonance
DW: diffusion-weighted
ROI: region of interest
CTV: clinical target volume
PTV: planning target volume
ICC: intraclass correlation coefficient
ROC: receiver operating characteristic
